# Reinforcement Learning-Based End-to-End Parking for Automatic Parking System

**DOI:** 10.3390/s19183996

**Published:** 2019-09-16

**Authors:** Peizhi Zhang, Lu Xiong, Zhuoping Yu, Peiyuan Fang, Senwei Yan, Jie Yao, Yi Zhou

**Affiliations:** 1 School of Automotive Studies, Tongji University, Shanghai 201804, China; zhangpeizhi@tongji.edu.cn (P.Z.); yuzhuoping@tongji.edu.cn (Z.Y.); funpayyuan@foxmail.com (P.F.); 2 SAIC Motor Corporation Limited, Shanghai 201800, China; adam_yan@foxmail.com (S.Y.); yaojie@saicmotor.com (J.Y.); zhouyi02@saicmotor.com (Y.Z.)

**Keywords:** automatic parking system (APS), end-to-end parking, reinforcement learning, parking slot tracking

## Abstract

According to the existing mainstream automatic parking system (APS), a parking path is first planned based on the parking slot detected by the sensors. Subsequently, the path tracking module guides the vehicle to track the planned parking path. However, since the vehicle is non-linear dynamic, path tracking error inevitably occurs, leading to inclination and deviation of the parking. Accordingly, in this paper, a reinforcement learning-based end-to-end parking algorithm is proposed to achieve automatic parking. The vehicle can continuously learn and accumulate experience from numerous parking attempts and then learn the command of the optimal steering wheel angle at different parking slots. Based on this end-to-end parking, errors caused by path tracking can be avoided. Moreover, to ensure that the parking slot can be obtained continuously in the process of learning, a parking slot tracking algorithm is proposed based on the combination of vision and vehicle chassis information. Furthermore, given that the learning network output is hard to converge, and it is easy to fall into local optimum during the parking process, several reinforcement learning training methods in terms of parking conditions are developed. Lastly, by the real vehicle test, it is proved that using the proposed method can achieve a better parking attitude than using the path planning and path tracking-based method.

## 1. Introduction

The average proportion of cars and parking slots in big cities is about 1:0.8, and that in small and medium-sized cities is nearly 1:0.5, according to the data released by the National Development and Reform Commission of China. The lack of parking space makes the designed parking slot increasingly narrower. Accordingly, parking environment is becoming complex progressively, and the increasingly higher requirement of the parking operation accuracy is raised, bringing great troubles to many drivers. Automatic parking system (APS) can increase parking safety and utilization rate of parking slot, so it has wide market application prospects.

However, the smaller size of the parking slot requires very high parking accuracy for APS. Take the perpendicular parking slot as an example; it raises a higher demand of parking attitude for its narrow width. The BS ISO 16787-2016 [1] stipulates that the perpendicular parking inclination angle of APS should be confined within ±3°, imposing huge challenges to the performance of APS.

The current mainstream APS architecture is a path planning and path tracking-based method. To be specific, a parking path is first planned based on the parking slot detected by the sensors (e.g., camera and ultrasonic radar), and then the path tracking module controls the vehicle to track the planned parking path. However, since the vehicle is nonlinear dynamic, the control error of path tracking is inevitable during the path tracking, leading to inappropriate parking attitude.

How can we avoid the path tracking error to ensure the ideal parking attitude? Let us think about how humans park their cars. Actually, we directly turn the steering wheel according to the position of the parking slot, which is an end-to-end parking mode. Moreover, as the number of parking increases, we gain more experience, and parking is increasingly accurate. In fact, reinforcement learning is an algorithm in which the agent gets the greatest reward in the process of interactive learning with the environment, thus learning the optimal mapping from environment to action. Therefore, we try to apply reinforcement learning to APS to improve the parking attitude.

### 1.1. Related Work

#### 1.1.1. Mainstream APS

The mainstream APS first plans a parking path according to the parking slot detected by sensors. Path planning can be split into geometric method, sampling method, and numerical optimization method. The geometric method adopts Reeds–Shepp (RS) curve [2], B-spline curve [3], η^3^-splines [4], and arcs optimized by cyclotron curve [5,6] to plan parking path based on the non-holonomic constraints of the vehicle. The sampling method aims to spread the points evenly in the sampling space, filter the points by a certain method, and connect the selected points into the required path, covering Rapidly-exploring Random Tree (RRT) [7] and target bias RRT [8]. The numerical optimization method is to consider the parking process as a dynamic system, and the length [9,10] or curvature [11,12] of the parking path is the optimization goal of this dynamic system. The constraints of this dynamic system include the non-holonomic constraints of the vehicle, the starting point, and the target location of the parking.

After completing the path planning, the path tracking module of APS controls the vehicle to track the planned parking path. Path tracking can be divided into Ackerman steering model-based open loop control method and vehicle dynamics model-based closed loop control method. The Ackerman steering model-based open loop control method considers that there is no tire sideslip, and vehicle satisfies the non-holonomic constraints. The most typical one is the pure tracking control algorithm [13]. The vehicle dynamics model-based closed loop control method considers tire sideslip. Feedforward control is designed using the two-degree-of-freedom vehicle dynamics model, and closed-loop feedback control is implemented by proportional-integral-differential (PID) algorithm [14,15] or sliding mode control (SMC) algorithm [16,17]. In fact, no matter which control method is used, the control error of the path tracking is inevitable since the vehicle is nonlinear dynamic [18,19], which makes the vehicle unlikely to completely track the planned parking path. Though there have been studies to reduce the path tracking error [20,21], the path tracking error cannot be eliminated.

#### 1.1.2. Reinforcement Learning

As mentioned above, path planning and path tracking-based method may result in poor parking attitude due to the inevitable control error (the experimental results in Section 3 also confirmed this). Accordingly, we take the “human-like” parking mode based on reinforcement learning, which cannot only avoid the error caused by path tracking through the end-to-end method of environment-to-action but also continuously learn and accumulate experience from considerable parking attempts, as well as learning the optimal steering wheel angle command at different parking slots relative to vehicle. How to choose a suitable reinforcement learning method for APS? To answer this question, different reinforcement learning methods are first reviewed.

Reinforcement learning mainly includes value-based method, policy-based method, and Actor-Critic method.

The value-based method evaluates the cumulative expectation reward by the value function after taking action and then chooses the action with the largest cumulative reward expectation [22]. Deep Q network (DQN) [23,24] is a typical value-based method. It is based on Q-learning and replaces Q-table with deep neural network (DNN) to solve the problem that Q-learning is prone to dimension disasters when state space is high-dimensional. However, value-based method makes value function continuous and chooses action based on the value function of each action, so it is not suitable for continuous action spaces (e.g., continuous steering wheel angle command for APS).

Compared with the value-based method, the policy-based method directly optimizes the policy based on the sampling method and constantly calculates the gradient of the policy expectation reward about the parameters of the policy network during the training process [25]. Though the policy-based method is applicable to high-dimensional continuous action spaces, each iterative step should sample a batch sequence to update the parameters, resulting in a large variance of the policy gradient estimation and making it easy to fall into local optimum.

The Actor-Critic method, which combines the value-based method and the policy-based method, adopts policy-based method to update the policy, and adopts the value function as the evaluation method of the policy [26,27,28]. By introducing the value function as the evaluation criterion in the policy search, the loss of sequential difference about the reward can be minimized, so that the variance of the policy gradient estimation can be reduced effectively. Although Actor-Critic method can realize the learning of continuous action space and can reduce the variance of the strategy gradient estimation, it only has one actor network and one critic network, which easily leads to unstable training. Deep Deterministic Policy Gradient (DDPG) algorithm [29,30] has made some improvements on the basis of Actor-Critic method. On one hand, it creates target networks for actor network and critic network, respectively, significantly enhancing the stability of learning. On the other hand, it uses experience pool-based replay caching technology to cut off the data correlation.

As mentioned above, we believe that DDPG is applicable to APS for the following reasons: first, Actor-Critic architecture can realize the learning of continuous action space (since the steering wheel angle for APS is a continuous action). Second, introducing the value function as the evaluation criterion in the policy search can reduce the variance of the policy gradient estimation, which is more efficient. Lastly, DDPG creates target networks for actor network and critic network, respectively, which makes it closer to the supervised learning and significantly enhance the stability of learning.

### 1.2. Objectives and Contributions

In brief, the current path planning and path tracking-based method cannot easily ensure the ideal parking attitude, especially the perpendicular parking slot, for its narrow width, which requires a higher demand for parking. To solve this problem, a reinforcement learning-based end-to-end parking algorithm is proposed in this paper for perpendicular parking. The main contributions are as follows:

- We innovatively apply DDPG to perpendicular parking so that the vehicle can continuously learn and accumulate experience from considerable parking attempts, learn the optimal steering wheel angle command at different parking slots relative to vehicle, as well as achieve the real “human-like” intelligent parking. Moreover, because it realizes the end-to-end control from the parking slot to the steering wheel angle command, the control errors caused by path tracking are fundamentally avoided;
- Since the parking slot needs to be continuously obtained in the course of learning, we propose a parking slot tracking algorithm, which uses extended Kalman filter (EKF) to fuse the parking slot information with vehicle chassis information to achieve continuous tracking of parking slot;
- Given that the learning network output is hard to converge and it is easy to fall into local optimum in the parking process, several reinforcement learning training methods in terms of parking conditions, e.g., manual guided exploration for accumulating initial experience sequence, control cycle phased setting, and training condition phased setting, are designed. Besides, the well-trained network in the simulation environment is migrated to the real vehicle training.

### 1.3. Paper Outline

The rest of this paper is organized as follows. In Section 2, our reinforcement learning-based end-to-end parking method is introduced. In Section 3, the experimental results are showed. In Section 4, some discussions are contained. Lastly, this paper is concluded in Section 5.

## 2. Method

**Figure 1:**
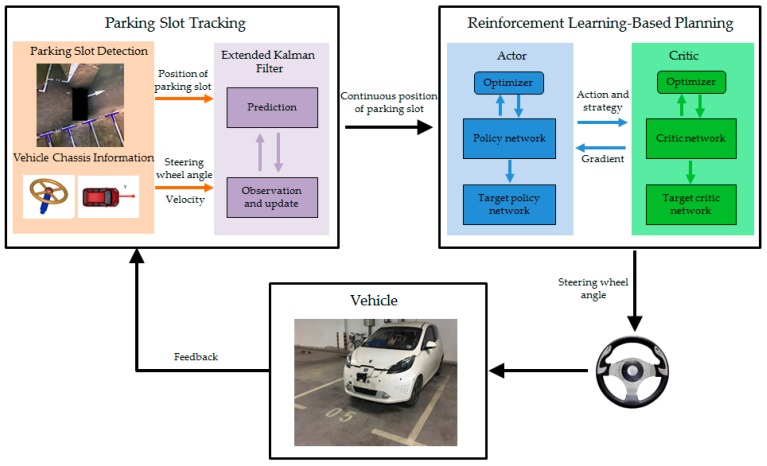
Overview of the reinforcement learning-based end-to-end parking method.

The overview of the proposed method is shown in Figure 1. It primarily includes two modules, parking slot tracking and reinforcement learning-based planning. Parking slot tracking is used to provide continuous position of parking slot for reinforcement learning, and reinforcement learning is adopted to achieve end-to-end planning from the parking slot to steering wheel angle.

### 2.1. Parking Slot Tracking

In this section, the parking slot detection is first introduced, followed by the EKF-based parking slot tracking.

#### 2.1.1. Parking Slot Detection

**Figure 2:**
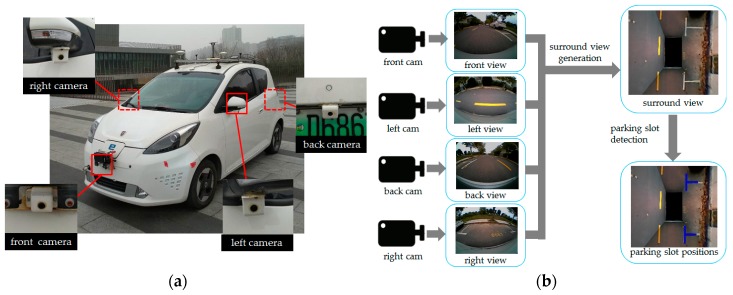
Surround view parking slot detection system. (**a**) Test vehicle and camera installation location. (**b**) Surround view generation and parking slot detection.

The sensors of surround view parking slot detection system are outfitted with four fisheye cameras in the front, rear, left, and right positions of the vehicle, respectively, with 180° of FOV horizontally and 140° of FOV vertically, as shown in Figure 2a.

The parking slot detection consists of two steps: one is to yield a surround view based on the images taken by the four fisheye cameras; the other is to detect the corner points of parking slots using the surround view. The flow chart is shown in Figure 2b.

**Figure 3:**
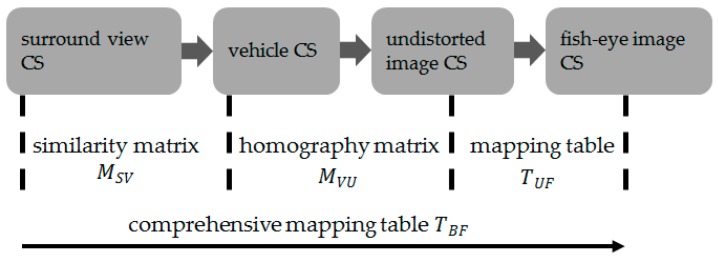
Surround view generation process.

For the generation of surround view, first, the distortion parameters of fisheye camera are calculated by Zhang Zhengyou’s calibration method [31], and the mapping table $T_{UF}$ from undistorted image coordinate system (CS) to fisheye image CS is yielded. Subsequently, based on the checkerboard calibration site, the homography matrix $M_{VU}$ from vehicle CS to undistorted image CS is calculated using the least square method. Lastly, after confirming the scope and the image size of the surround view, the similarity transformation matrix $M_{SV}$ of surround view CS to vehicle CS is calculated. Four fisheye perspectives are joined into one surround view by the comprehensive mapping table $T_{BF}$ constructed above. The whole process is illustrated in Figure 3.

**Figure 4:**
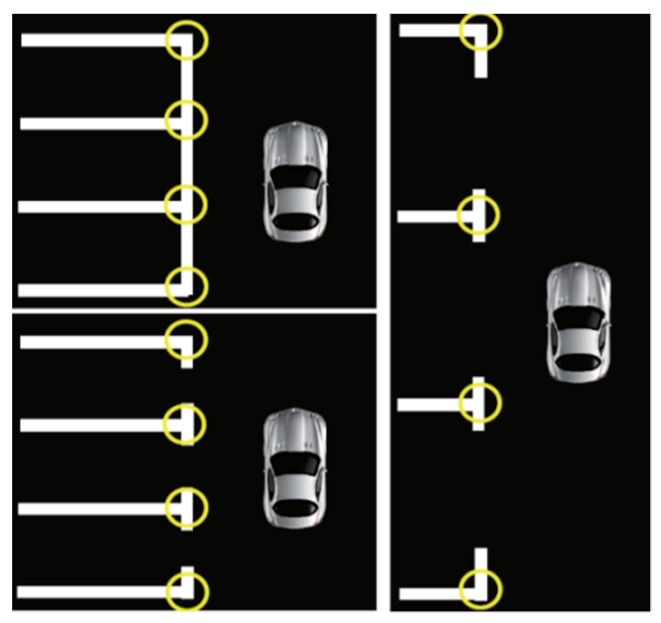
The “L” and “T” corner points.

The method proposed by Li et al. [32] is adopted to detect parking slot, which is an AdaBoost-based slot detection method that detects parking slots from surround view. This method is primarily used to detect common “L” and “T” corner points, as shown in Figure 4. The basic principle is to use Adaboost algorithm and decision tree to design a binary classifier to detect corner point patterns. The input of the classifier refers to an image patch, and the output is a Boolean value, indicating whether the input local block is a corner pattern. Because of the limited FOV of surround view, the length of the parking slot can be inferred following some prior rules after the detection of the corner points.

**Figure 5:**
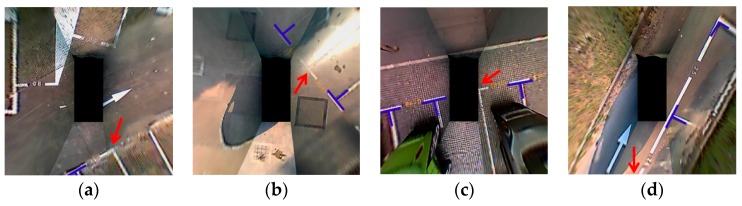
Missed parking slot detections (indicated by red arrows). (**a**) Image distortion. (**b**) Illumination change. (**c**) Occlusion. (**d**) Limited FOV.

The above analysis reveals that when detecting the corner points, the parking slot relative to the vehicle can be calculated through coordinate transformation. However, we find that the parking slot is difficult to continuously identify by relying solely on the vision. For instance, the corner points of the parking slot are sometimes not detected during parking due to image distortion, illumination change, occlusion, as well as the limited FOV, as shown in Figure 5.

#### 2.1.2. EKF-Based Parking Slot Tracking

To track parking slot continuously and accurately, EKF is employed to achieve the fusion of vision and vehicle chassis information. First, we take the position of the corner point of parking slot relative to vehicle as the EKF’s observation, building the constraint relationship between the position of vehicle and parking slot in the reference CS, i.e., the EKF’s observation model. Second, the vehicle kinematics model is taken as the EKF’s motion model, and the steering wheel angle and velocity obtained from vehicle chassis act as the EKF’s control input. Lastly, based on EKF’s “prediction” and “update” process, the fusion is completed to achieve the maximum posterior estimation of parking slot in the presence of noise.

**Figure 6:**
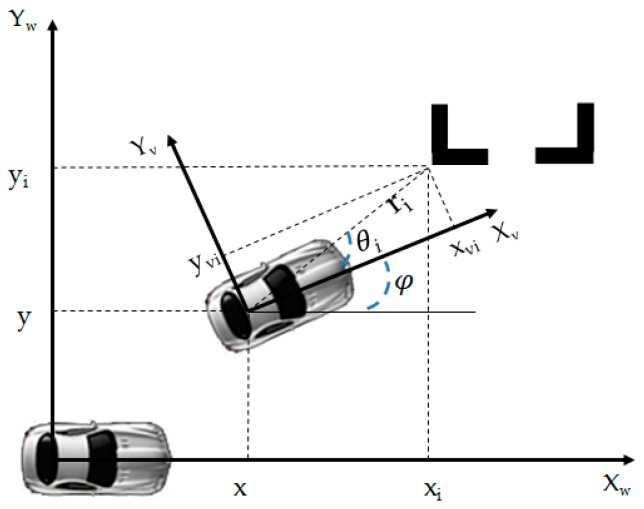
CS and parameter definition.

The definition of CS and parameters is shown in Figure 6, where (x,y), φ and $\left(x_{i},y_{i}\right)$ are the vehicle coordinates, vehicle heading angle and the i th corner points (i = 1 and 2 represent the left and right corner point of parking slot, respectively) coordinates in the reference CS, respectively. $\left(x_{vi},y_{vi}\right)$ obtained by parking slot detection system in Section 2.1.1 denotes the coordinates of the i th corner point in the vehicle CS. Its distance from the center of the rear axle of the vehicle is $r_{i}$, and its angle relative to the axis of the vehicle CS is $\theta_{i}$. $r_{i}$ and $\theta_{i}$ can be derived from the Equation (1).

(1) $$\begin{array}{c} \theta_{i}\left(k\right)=\arctan\left(\frac{y_{vi}\left(k\right)}{x_{vi}\left(k\right)}\right)\\ r_{i}\left(k\right)=\sqrt{x_{vi}{\left(k\right)}^{2}+y_{vi}{\left(k\right)}^{2}} \end{array}\;$$

The state variable of EKF is $X={\left(x,y,\varphi ,x_{i},y_{i}\right)}^{T}$, and its covariance matrix of the error is denoted as P. The observed variable of the system is expressed as $Z={\left(r_{i},\theta_{i}\right)}^{T}$.

The discrete observation model is expressed as Equation (2),

(2) $$\begin{array}{c} Z\left(k\right)=h\left(X\left(k\right)\right)+\vartheta \left(k\right)\\ \left[\sqrt{\begin{array}{c} {\left(x_{i}\left(k\right)-x\left(k\right)\right)}^{2}+{\left(y_{i}\left(k\right)-y\left(k\right)\right)}^{2}\\ \arctan\left(\frac{y_{i}\left(k\right)-y\left(k\right)}{x_{i}\left(k\right)-x\left(k\right)}\right)-\varphi \left(k\right) \end{array}}\right]+\vartheta \left(k\right) \end{array}\;$$

where ϑ(k) denotes the noise of parking slot detection system, assuming a Gaussian distribution; its covariance matrix is R.

According to the Ackerman steering model of the vehicle, the discrete motion model can be expressed as Equation (3),

(3) $$\begin{array}{c} X\left(k\right)=f\left(X\left(k-1\right),U\left(k\right)\right)+w\left(k\right)\\ =\left[\begin{array}{c} x\left(k-1\right)+Tv\left(k\right)cos\varphi \left(k\right)\\ y\left(k-1\right)+Tv\left(k\right)sin\varphi \left(k\right)\\ \varphi \left(k-1\right)+\frac{Tv\left(k\right)\tan\left(\delta \left(k\right)/i_{0}\right)}{L}\\ x_{i}\left(k-1\right)\\ y_{i}\left(k-1\right) \end{array}\right]+w\left(k\right) \end{array}\;$$

where δ denotes the steer wheel angle; v the velocity; δ and v can be obtained directly from the vehicle chassis; $i_{0}$ the steering gear ratio; L the wheelbase; T the period; w(k) the noise of the motion model, assumed to be Gaussian noise, and its covariance matrix is expressed as Q.

EKF can be split into two steps (prediction and update). First, the system state and its error covariance matrix at the k th iteration time are predicted, as expressed in Equation (4),

(4) $$\begin{array}{c} \hat{X}{\left(k\right)}^{-}=f\left(\hat{X}\left(k-1\right),U\left(k\right)\right)\\ P{\left(k\right)}^{-}=F\left(k\right)P\left(k-1\right)F{\left(k\right)}^{T}+Q \end{array}\;$$

where F(k) denotes the Jacobian of function f(X(k),U(k)) with respect to X(k).

Subsequently, it is the update process. First, the Kalman gain K(k) is calculated, which is the key to the maximum posteriori estimation of X(k) in the presence of noise, as shown in Equation (5). Second, X(k) and P(k) are updated by K(k), as expressed in Equation (6),

(5) $$K\left(k\right)=P{\left(k\right)}^{-}H{\left(k\right)}^{T}{\left(H\left(k\right)P{\left(k\right)}^{-}H{\left(k\right)}^{T}+R\right)}^{-1}\;$$

(6) $$\begin{array}{c} \hat{X}\left(k\right)=\hat{X}{\left(k\right)}^{-}+K\left(k\right)\left[Z\left(k\right)-H\left(k\right)\hat{X}{\left(k\right)}^{-}\right]\\ P\left(k\right)=\left(I-K\left(k\right)H\left(k\right)\right)P{\left(k\right)}^{-} \end{array}\;$$

where H(k) is the Jacobian of function h(X(k)) with respect to X(k).

Equation (6) reveals that K(k) helps fuse the vision and vehicle chassis information, and the updated $\hat{X}\left(k\right)$ is calculated by K(k) in the presence of noise, satisfying the maximum posterior estimate of X(k). Accordingly, EKF-based fusion is more accurate than relying solely on vision detection. Moreover, X(k) is continuous because the vehicle chassis information continues to be inputted. Thus, the continuous and accurate position of the parking slot relative to the vehicle can be derived from the above.

### 2.2. Reinforcement Learning-Based Planning

In this section, reinforcement learning is adopted to achieve end-to-end planning from the parking slot to steering wheel angle. We first introduce the appropriate reinforcement learning model for APS, followed by the system settings and training process of DDPG, and finally the improved training measures applied in parking.

#### 2.2.1. Appropriate Reinforcement Learning Model for APS

The basic process of reinforcement learning is a Markov decision-making process, which can be expressed by the quaternion {S,A,P,R} composed of state S, action A, state transition probability P and reward R.

When a policy π is executed at time t, cumulative reward $G_{t}$ can be calculated:

(7) $$G_{t}=R_{t}+\gamma R_{t+1}+\gamma^{2}R_{t+2}+\cdots =\sum_{k=0}\gamma^{k}R_{t+k+1}$$

where γ is the discount factor, which is used to reduce the reward weight corresponding to the long-term decision.

The action value function $Q_{\pi }\left(s,a\right)$ is the expectation of the cumulative reward $G_{t}$ after taking action a at the current state s, as expressed in Equation (8).

(8) $$Q_{\pi }\left(s,a\right)=E_{\pi }[G_{t}|S_{t}=s,A_{t}=a]$$

The action valued function $Q_{\pi }\left(s,a\right)$ satisfies the Bellman equation (Equation (9)), which transforms the solution of $Q_{\pi }\left(s,a\right)$ into an iterative process of dynamic programming.

(9) $$Q_{\pi }\left(s,a\right)=E_{\pi }[R_{t+1}+\gamma q_{\pi }\left(S_{t+1},A_{t+1}\right)|S_{t}=s,A_{t}=a]$$

The goal of reinforcement learning is to find an optimal policy for obtaining the maximum $Q_{*}\left(s,a\right)$, as shown in the following equation.

(10) $$Q_{*}\left(s,a\right)=\underset{\pi }{max}Q_{\pi }\left(s,a\right)$$

According to the different optimization objects, reinforcement learning methods can be divided into value-based method, policy-based method, and Actor-Critic method.

• Value-based method

Q-learning is a basic value-based method. Q-learning first chooses action a according to Q value at the current state s in each step of the cycle (e.g., using ε−greedy method: 1−ε probability of selecting action $\arg\underset{a}{\max}Q\left(s,a\right)$, ε probability of randomly selecting action). After the selected action is taken, the immediate reward R and the next state $s^{\prime }$ are observed, and then Q(s,a) is updated, as expressed in Equation (11). Repeat the process until the final state is reached,

(11) $$Q\left(s,a\right)\leftarrow Q\left(s,a\right)+\alpha \left[R+\gamma \;\underset{a}{\max}\;Q\left(s^{\prime },a\right)-Q\left(s,a\right)\right]$$

where α is the learning rate.

DQN replaces Q-table with DNN with parameter w (Equation (12)) to solve the problem that Q-learning is prone to dimension disasters when state space is high-dimensional.

(12) $$Q_{w}\left(s,a\right)\approx Q\left(s,a\right)$$

The updating objective of $Q_{w}\left(s,a\right)$ is to minimize the mean square deviation of the objective value $Q_{\pi }\left(s,a\right)$ and the actual value $Q_{w}\left(s,a\right)$, as shown in Equation (13). If gradient descent method is used, the gradient $\nabla_{w}J\left(w\right)$ of the objective function J(w) relative to the parameter w is first calculated, and then the parameter w changes along the opposite direction of the gradient $\nabla_{w}J\left(w\right)$, as shown in Equation (14),

(13) $$J\left(w\right)=E_{\pi }\left[{\left(Q_{\pi }\left(s,a\right)-Q_{w}\left(s,a\right)\right)}^{2}\right]$$

(14) $$w\leftarrow w+\alpha \nabla_{w}J\left(w\right)=w+\alpha \left[R+\gamma Q_{w}\left(s^{\prime },a^{\prime }\right)-Q_{w}\left(s,a\right)\right]\nabla Q_{w}\left(s,a\right)$$

where $Q_{\pi }\left(s,a\right)$ can be solved by temporal-difference method, i.e., $Q_{\pi }\left(s,a\right)=R+\gamma Q_{w}\left(s^{\prime },a^{\prime }\right)$.

Since the action space of the value-based method is discrete, it is not suitable for the continuous action space of parking control. Though the continuous action space can be discretized, too large discrete spacing will lead to the algorithm not getting the optimal action, and too small discrete spacing will lead to dimension disaster.

• Policy-based method

The policy-based method directly optimizes the policy based on the sampling method, and constantly calculates the gradient $\nabla_{\theta }J\left(\theta \right)$ of the policy expectation reward J(θ) about the policy parameter θ during the training process, as expressed in Equations (15) and (16),

(15) $$J\left(\theta \right)=E_{\pi_{\theta }}\left[R\right]=\sum_{s\in S}d^{\pi }\left(s\right)\sum_{a\in A}\pi_{\theta }\left(s,a\right)R_{s,a}$$

(16) $$\nabla_{\theta }J\left(\theta \right)=\underset{s\in S}{\sum }d^{\pi }\left(s\right)\underset{a\in A}{\sum }\pi_{\theta }\left(s,a\right)\nabla_{\theta }log\pi_{\theta }\left(s,a\right)R_{s,a}=E_{\pi_{\theta }}\left[\nabla_{\theta }log\pi_{\theta }\left(s,a\right)R_{s,a}\right]$$

where $\pi_{\theta }\left(s,a\right)$ is the policy of action selection, which represents the probability of choosing action a under the state s; $d^{\pi }\left(s\right)$ is the static distribution of the state s under the policy π.

If the Monte Carlo policy gradient algorithm is used, the iteration equation of the policy parameter θ is as follows:

(17) $$\theta \leftarrow \theta +\alpha \nabla_{\theta }\;log\pi_{\theta }\left(s,a\right)v$$

where v is equal to the cumulative reward $G_{t}$ of the current step minus the average cumulative reward $\left(1/T\right)\sum_{t=1}^{T}G_{t}$, i.e., if the action gets better evaluation than before and v is positive, it will increase the probability of this action being selected.

Though the policy-based method is applicable to high-dimensional continuous action spaces, each iterative step should sample a batch sequence to update the parameters, resulting in a large variance of the policy gradient estimation and making it easy to fall into local optimum.

• Actor-Critic method

The Actor-Critic method, which combines the value-based method and the policy-based method, consists of two updating processes: The critic network is responsible for updating the network parameters of the action value function, observing the action and reward, and evaluating the policy. The actor network is responsible for updating the actor network parameters according to the guidance of the critic networks. By introducing the value function as the evaluation criterion in the policy search, the loss of sequential difference about the reward can be minimized so that the variance of the policy gradient estimation can be reduced effectively.

Although Actor-Critic method can realize the learning of continuous action space and can reduce the variance of the policy gradient estimation, it only has one policy network and one critic network, which easily leads to unstable training.

In order to solve this problem, DDPG constructs the target network with parameter $\theta^{\prime }$, which is used to calculate the target value. The target network is adopted to track the actor network and critic network slowly to update the parameter $\theta^{\prime }$, as expressed in Equation (18). This means that the target value is limited to slow change, which greatly improves the stability of learning. This improvement brings the reinforcement learning closer to supervised learning.

(18) $$\theta^{\prime }\leftarrow \tau \theta +\left(1-\tau \right)\theta^{\prime },\tau \leq 1$$

In addition, a challenge in reinforcement learning using neural networks is that most optimization algorithms assume that the samples are independent and identically distributed. Obviously, this assumption is no longer valid when the samples are sequentially explored in the environment. DDPG uses a finite size experience pool to cut off the data correlation. The experience sequence is sampled from the environment according to the exploratory strategy, and the tuples are stored in the experience pool. When the experience pool is full, discard the oldest sample. At each time step, the actor network and critic network are updated by uniformly sampling small batches from the experience pool.

As mentioned above, we believe that DDPG is applicable to APS for the following reasons: First, Actor-Critic architecture can realize the learning of continuous action space (since the steering wheel angle for APS is a continuous action). Second, introducing the value function as the evaluation criterion in the policy search can reduce the variance of the policy gradient estimation, which is more efficient. Third, DDPG creates target networks for actor network and critic network, respectively, which makes it closer to the supervised learning and significantly enhances the stability of learning. Lastly, the experience pool is adopted to cut off the data correlation.

#### 2.2.2. System Settings of DDPG

In this section, we mainly introduce the system settings of DDPG, including input and output, reward and network.

• Input and output

The input state s of DDPG refers to the parking slot relative to the vehicle, i.e., the coordinates of the four corner points in the vehicle CS. The output action a of DDPG is the steering wheel angle, capable of controlling the vehicle backing into the parking slot.

• Reward

At present, the reward of reinforcement learning mainly depends on expert experience. The goal of proposed algorithm is to make the vehicle parked in the middle of the parking slot, avoiding inclination, deviation, and line-pressing. We take these factors into consideration and through a large number of simulation training get a better reward setting as shown in Equations (19) to (24).

The total reward R consists of three parts, as expressed in Equation (19). The first part $R_{cp}$ considers the reward that the vehicle tends to the center of the parking slot, and vehicle longitudinal axis parallels to the parking slot. The second part $P_{l}$ and the last part $P_{d}$ consider the punishment of line-pressing and the punishment of the vehicle’s deviation to one side of the parking slot, respectively.

(19) $$R=R_{cp}+P_{l}+P_{d}\;$$

**Figure 7:**
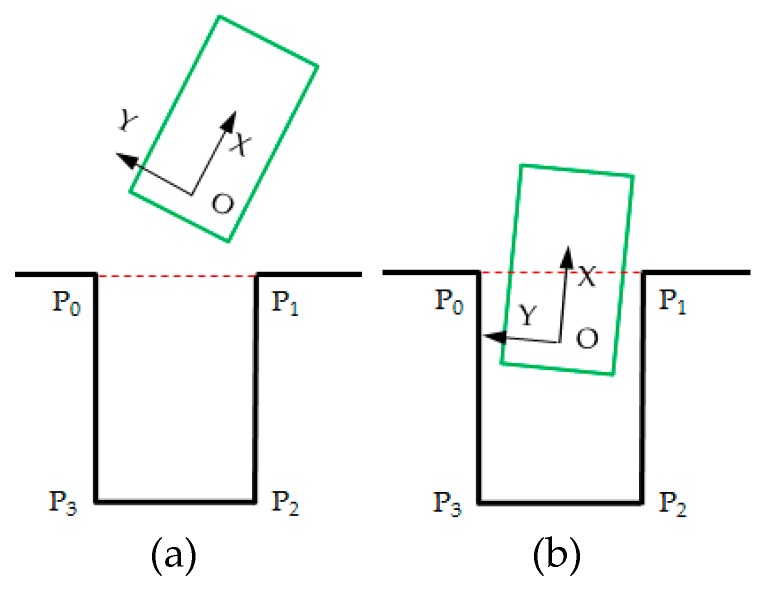
Different parking stages. (**a**)The rear axle center of the vehicle is outside the outer line of the parking slot. (**b**) The rear axle center of the vehicle crosses the outer line of the parking slot.

As shown in Figure 7a, when the rear axle center of the vehicle is outside the outer line of the parking slot, $P_{cp}$ is defined as:

(20) $$\begin{array}{c} P_{cp}=P_{c}+P_{p}\\ =\left(5-5\left(\frac{1}{2}abs\left(Y_{p_{0}}+Y_{p_{1}}\right)+\frac{1}{2}abs\left(Y_{p_{2}}+Y_{p_{3}}\right)\right)\right)+\left(5-5abs\left(\frac{Y_{p_{0}}-Y_{p_{3}}}{X_{p_{0}}-X_{p_{3}}}\right)\right) \end{array}\;$$

where $P_{c}$ denotes the reward for the vehicle to be close to the center of the parking slot; $P_{p}$ the reward for the vehicle’s longitudinal axis parallel to the parking slot; (X,Y) the coordinates of the corner points ($P_{0}-P_{3}$ in Figure 7) of the parking slot in the vehicle CS.

As shown in Figure 7b, when the rear axle center of the vehicle crosses the outer line of the parking slot, $R_{cp}$ is defined as:

(21) $$R_{cp}=min\left\{R_{c},R_{p}\right\}+\frac{1}{2}max\left\{R_{c},R_{p}\right\}+R_{n}\;$$

The reason why the larger values in $R_{c}$ and $R_{p}$ are reduced is to prevent falling into one better and conceal the worse performance of the other, so more attention is paid to the worse performance of the two. Besides, when the vehicle enters the parking slot, we pay more attention to the parallelism between the vehicle and the parking slot. Accordingly, a reward $R_{n}$ is set, which is expressed in Equation (22). It is limited to a value of less than 10 to avoid covering other rewards.

(22) $$R_{n}=abs(\frac{1}{10}min\{1/abs\left(\frac{Y_{p_{0}}-Y_{p_{3}}}{X_{p_{0}}-X_{p_{3}}}\right)+eps),100\}))\;$$

If any outer contour boundary of the vehicle intersects with the parking slot lines, it is considered a line-pressing, $P_{l}$ is defined as:

(23) $$P_{l}=-10$$

If the vehicle is biased towards one side of the parking slot, $P_{d}$ is defined as:

(24) $$P_{d}=-10$$

• Network

The input of our network is not the image but the result of the parking slot detection. Thus, the deep neural networks are not necessarily required to be used. For actor network and critic network of DDPG, we just use back propagation neural network in this paper. Besides, we build the target network with an identical structure but different parameters for actor network and critic network and the relationship between network parameter θ and its target network parameters $\theta^{\prime }$ is $\theta^{\prime }\leftarrow \tau \theta +\left(1-\tau \right)\theta^{\prime },\tau \ll 1$, significantly enhancing the stability of learning.

**Figure 8:**
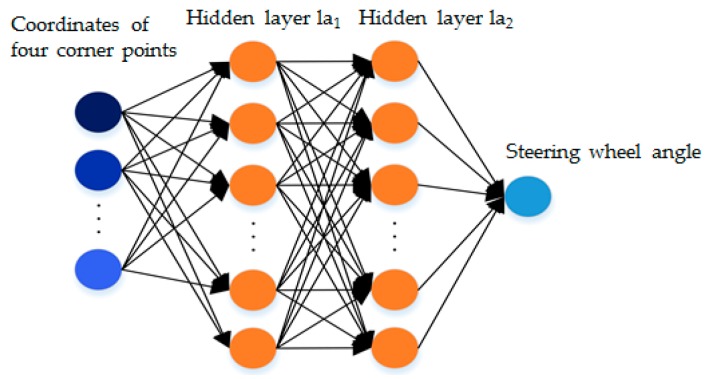
The structure of actor network and target actor network.

The structure of actor network and target actor network is illustrated in Figure 8. The number of nodes for the coordinates of four corner points is 8, and the number of nodes in hidden layer $la_{1}$ and hidden layer $la_{2}$ is 100 and 200, respectively. Then, the number of nodes for steering wheel angle is 1. All activation functions are Rectified Linear Unit (ReLU). 

**Figure 9:**
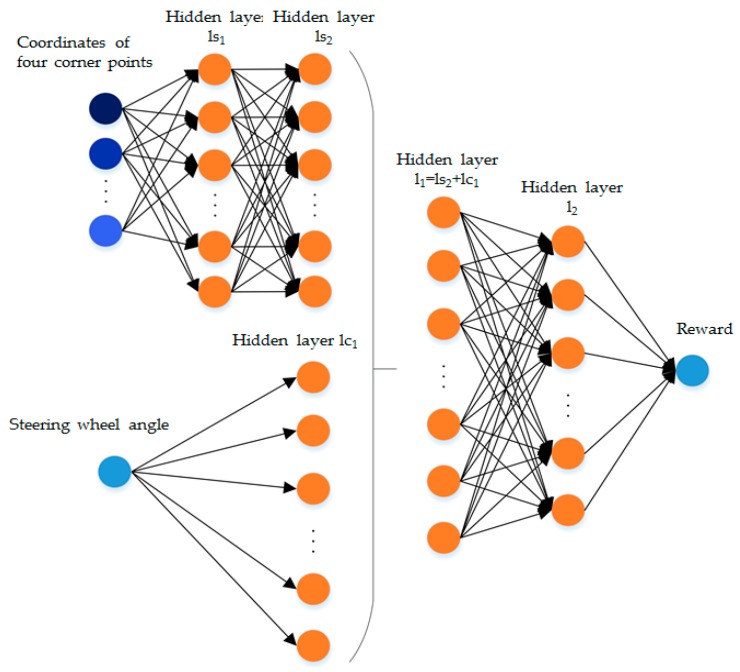
The structure of critic network and target critic network.

The structure of critic network and target critic network is shown in Figure 9. The number of nodes for the coordinates of four corner points is 8, and the number of nodes in hidden layer $ls_{1}$ and hidden layer $ls_{2}$ are both 100. The number of nodes for steering wheel angle is 1, and the number of nodes in hidden layer $lc_{1}$ is 200. Subsequently, the number of nodes in hidden layer $l_{1}$ and hidden layer $l_{2}$ is 300 and 200, respectively. Lastly, the number of nodes for reward is l. All activation functions are also ReLU.

#### 2.2.3. Training Process of DDPG

**Figure 10:**
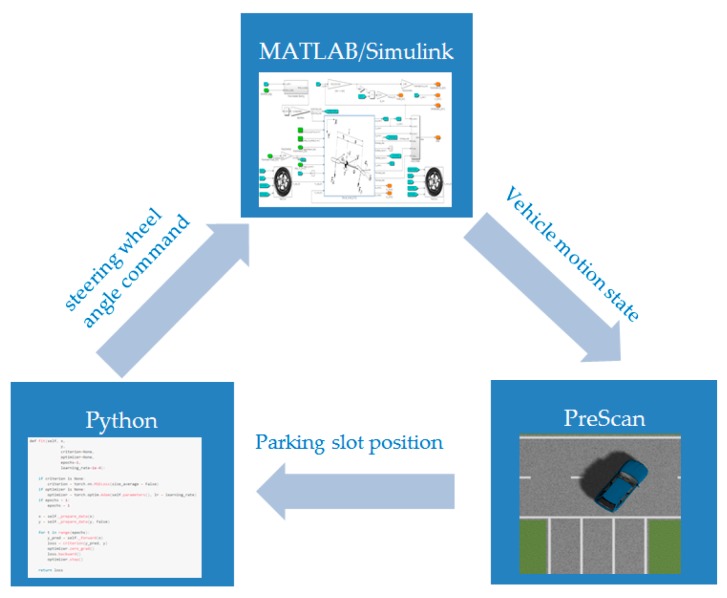
Simulation platform.

First, the training is conducted in the simulation environment. The simulation platform is shown in Figure 10. We use PreScan, MATLAB/Simulink, and Python in sequence to build the parking environment, build the vehicle model, and then run our algorithm, respectively. After the simulation training, the well-trained network migrates to the real vehicle training. In Section 3, the real vehicle platform will be introduced.

**Figure 11:**
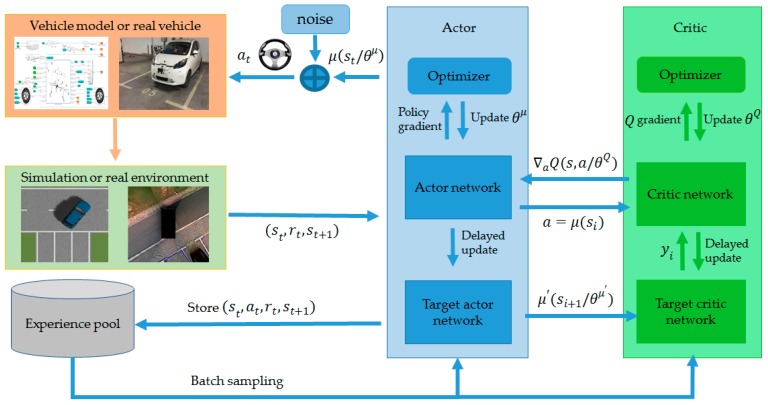
The training architecture of DDPG.

The training architecture of DDPG is shown in Figure 11, and the corresponding training process is shown in Algorithm 1.


**Algorithm 1: DDPG Algorithm**
Randomly initialize critic network $Q\left(s,a/\theta^{Q}\right)$ and actor network $\mu \left(s/\theta^{\mu }\right)$ with parameters $\theta^{Q}$ and $\theta^{\mu }$
Initialize target critic network $Q^{\prime }$ and target actor network $\mu^{\prime }$ with parameters $\theta^{Q^{\prime }}$ and $\theta^{\mu^{\prime }}$
Set up a replay memory buffer (experience pool) for the sampling experience sequence with the total number of buffers M **for** each episode: Initialize a random process for action exploration Receive initial state $s_{1}$ **for**
t=1,T:   Select action $a_{t}$ according to the current policy and exploration noise:
$$a_{t}=\mu \left(s_{t}/\theta^{\mu }\right)+N_{t}$$ where $N_{t}$ denotes Gaussian noise.Execute action $a_{t}$ and obtain the reward $r_{t}$ and the next state $s_{t+1}$
Store the transition $\left(s_{t},a_{t},r_{t},s_{t+1}\right)$ in the experience poolRandomly sample N experience sequences from experience pool as a mini-batch training data for the critic network and actor networkThis step is adopted to update the parameters of the critic network. With a method similar to supervised learning, loss is defined as: $$L=\frac{1}{N}\underset{i}{\sum }{\left(y_{i}-Q\left(s_{i},a_{i}/\theta^{Q}\right)\right)}^{2}$$ where $y_{i}$ is calculated based on $\mu^{\prime }$ and $Q^{\prime }$: $$y_{i}=R_{i}+\gamma Q^{\prime }\left(s_{i+1},\mu^{\prime }\left(s_{i+1}/\theta^{\mu^{\prime }}\right)/\theta^{Q^{\prime }}\right)$$ Calculate the gradient $\nabla_{\theta^{Q}}L$, and then update $\theta^{Q}$ with gradient descent method: $$\theta^{Q}=\theta^{Q}+\alpha \;\nabla_{\theta^{Q}}L$$ where α is the learning rate.After the critic network is updated, the actor network is updated using the policy gradient method: $$\nabla_{\theta^{\mu }}J\approx \frac{1}{N}\underset{i}{\sum }\nabla_{a}Q\left(s,a/\theta^{Q}\right){/}_{s=s_{i},a=\mu \left(s_{i}\right)}\nabla_{\theta^{\mu }}\mu \left(s/\theta^{\mu }\right){/}_{s_{i}}$$ Update $\theta^{\mu }$ with $\nabla_{\theta^{\mu }}J$ based on gradient descent method: $$\theta^{\mu }=\theta^{\mu }+\alpha \nabla_{\theta^{\mu }}J$$Update the target networks: $$\theta^{Q^{\prime }}\leftarrow \tau \theta^{Q}+\left(1-\tau \right)\theta^{Q^{\prime }}$$
$\theta^{\mu^{\prime }}\leftarrow \tau \theta^{\mu }+\left(1-\tau \right)\theta^{\mu^{\prime }}$ **end for** **end for**


#### 2.2.4. Improved Training Measures Applied in Parking

Given that the learning network output is hard to converge and it is easy to fall into local optimum in the parking process, several reinforcement learning training methods in terms of parking conditions are designed.

• Manual guided exploration for accumulating initial experience sequence

**Figure 12:**
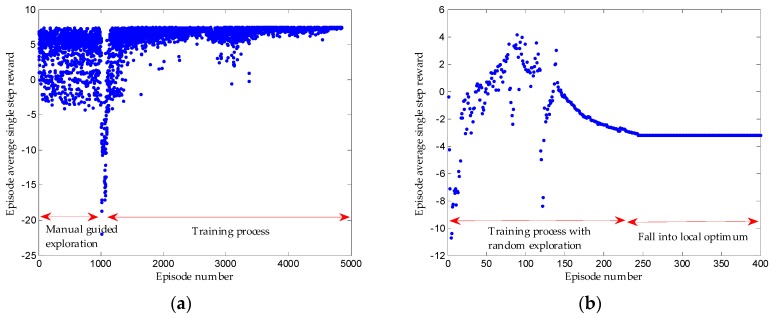
(**a**) Training process by manual guided exploration. (**b**) Training process by random exploration.

Before the training of network, exploration should be conducted to gain the initial experience sequence database. In the initialization stage, instead of random exploration, we conduct manual guidance on exploration, which is realized by setting a series of control commands for the initial parking slot relative to the vehicle (the driver’s control sequence is collected in the simulation or real vehicle test). Based on the manual control commands, the appropriate noise is added to give the model a better space for policy exploration and trial-and-error. In such a way, compared with random exploration, considerable experience sequences will receive higher rewards, which can make the training converge to excellent policy faster. The reward can converge eventually with manual guided exploration, as shown in Figure 12.

• Control cycle phased setting

**Figure 13:**
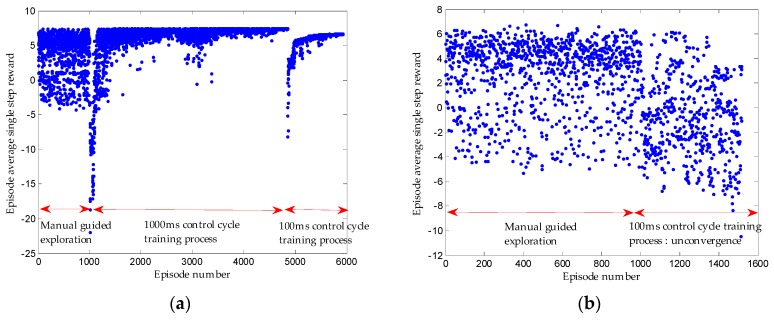
(**a**) Training process first by 1000 ms control cycle and followed by 100 ms control cycle. (**b**) Training process only by 100 ms control cycle.

Given that the vehicle model has inertia delay characteristics, it is found that if the period of steering wheel angle change is too small, it will cause the loss of Markov characteristics of some collected experience sequences. The state of the current cycle of the vehicle depends on both the state of the previous cycle and the action taken. To weaken this adverse effect, the first round of training sets the control cycle to 1000 ms. In such a way, the actions executed in the current cycle will retain sufficient execution time, which will be the major factor affecting the state of the next cycle and can be approximated to Markov decision-making process. When the network converges to the optimum, the training control cycle of the following training can be reduced, which can make the control cycle closer to the actual situation and achieve better results. Figure 13a shows that the 1000 ms control cycle is first trained, then the 100 ms control cycle is trained, and lastly the reward lastly converges. Figure 13b suggests that the reward does not converge if we start with a 100 ms control cycle training directly.

• Training condition phased setting

**Figure 14:**
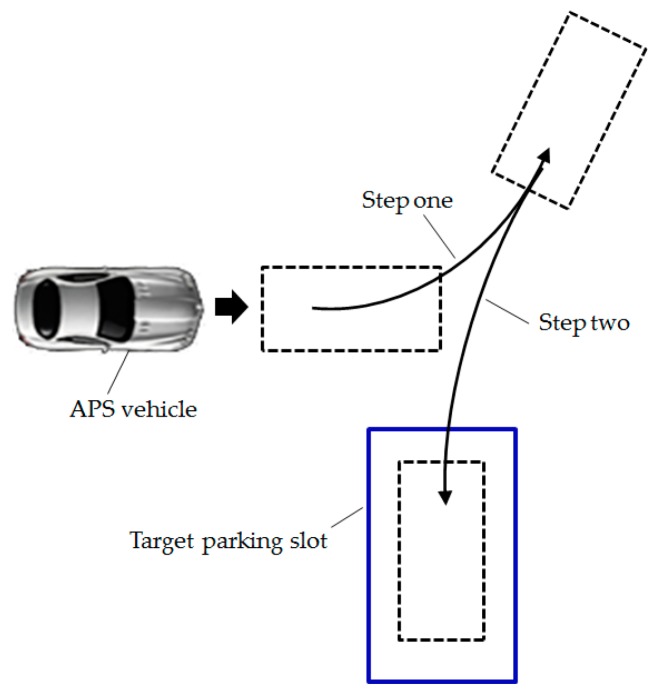
Common perpendicular parking process.

Usually the perpendicular parking can be split into two steps, as shown in Figure 14. Just like human parking, the “step two” plays a major role in the final parking attitude. Thus, we currently primarily apply reinforcement learning to the “step two”. 

**Figure 15:**
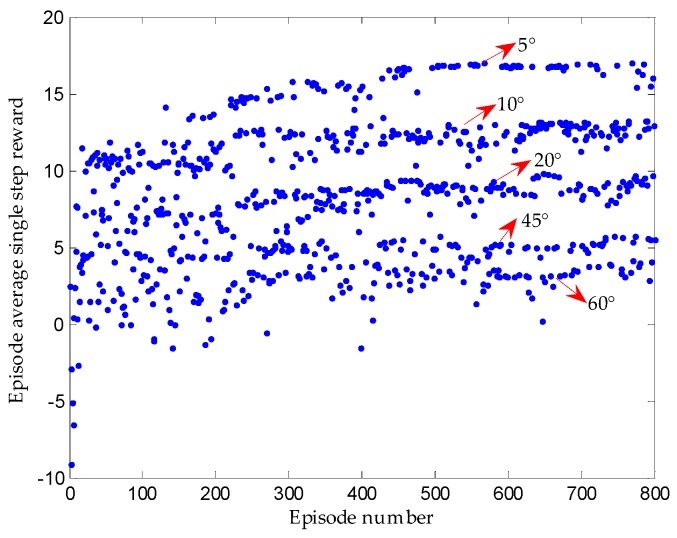
Extended training of other initial angles based on 30° well-trained network.

Since there will be different initial angles of “step two” between the vehicle and the parking slot, we first train at 30° and then expand the initial angle to 0° to 90° to continue training. Figure 15 suggests that based on 30° well-trained network, the networks between 0° to 90° can converge quickly, i.e., the 30° well-trained network has ideal generalization ability. Since the initial angle of the vehicle relative to the parking slot is different in different episodes, the sequence of states experienced in each episode is different, so the average single step reward is also different.

• Real vehicle training migration

**Figure 16:**
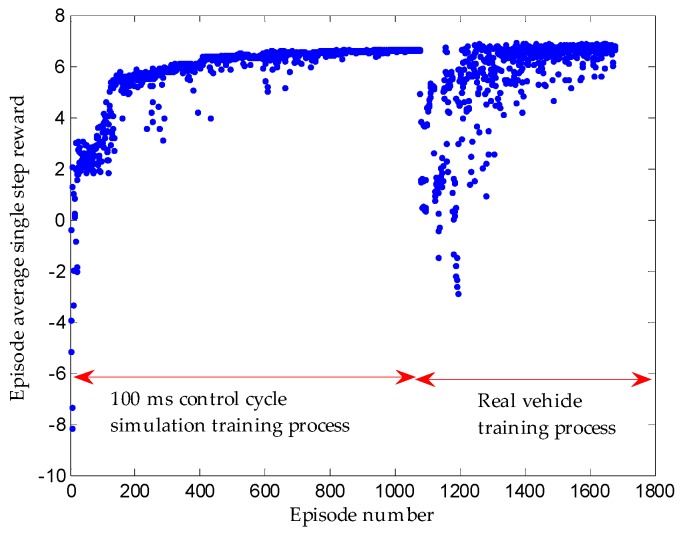
Real vehicle training migration.

Because the real vehicle training takes a lot of manpower, time and resources, it is better to train in the simulation environment and then transfer it to the real vehicle. Since the sensor model and vehicle model used in simulation will differ from the real vehicle, the same control command may produce different observation results. Accordingly, the real vehicle should be continuously trained based on well-trained network in simulation. Figure 16 reveals that the result of real vehicle migration training is ideal.

## 3. Experimental Results

After the above training, we can ascertain the performance of the trained algorithm. This section shows the experimental platform, experimental scenes, and results.

### 3.1. Experimental Platform

**Figure 17:**
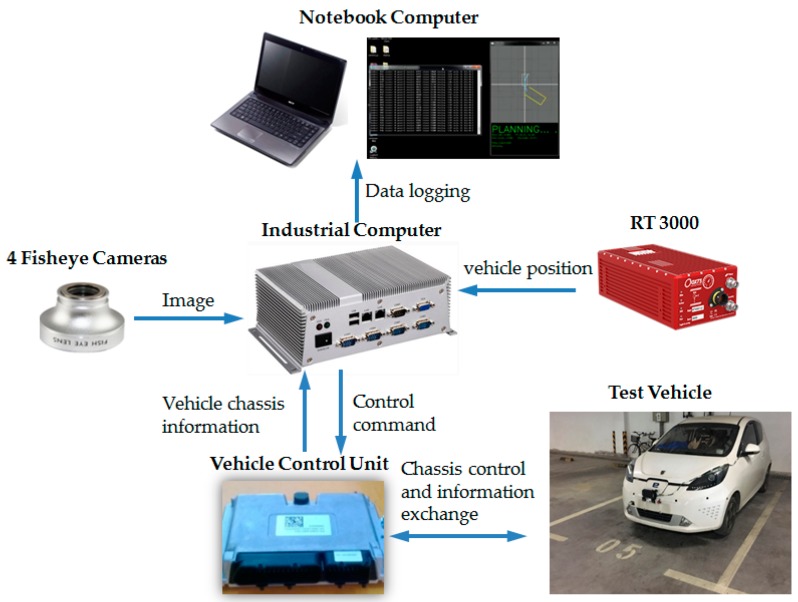
Experimental platform.

The experimental platform is refitted from Rongwei E50 pure electric vehicle (Figure 17). Four fisheye cameras act as sensors for parking slot detection. The algorithm running platform is an industrial computer (i5 processor, 8G memory, 128G solid-state hard disk). Chassis control and information exchange is performed in the vehicle control unit, i.e., the controller of vehicle chassis. The RT3000 navigation system is employed to acquire the position information of the vehicle. During the test, notebook computer is employed to record data.

### 3.2. Experimental Scenes

**Figure 18:**
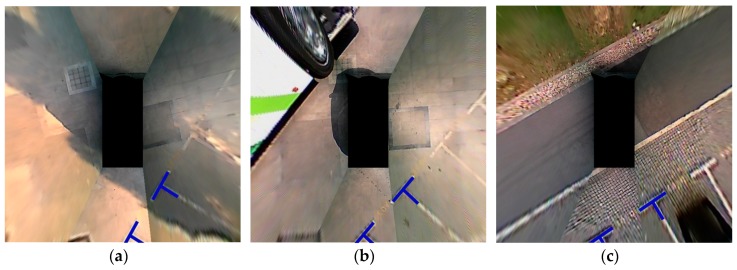
Experimental scenes. (**a**), (**b**) and (**c**) present parking scenes with initial angles of 60°, 45°, and 30°, respectively.

To ascertain the performance of the proposed algorithm in the “step two” perpendicular parking, we choose three parking scenes with initial angles of 60°, 45°, and 30° between the vehicle and the parking slot, which are common “step two” scenes. Figure 18 illustrates the experimental scenes expressed in the surround view. The blue marking points represent the target parking slots; the width of these parking slots ranges from 2.4 m to 2.44 m and the length is between 5.6 m and 5.8 m, which basically meets the test requirements of BS ISO 16787-2016. As described in Section 2.1.1, since the FOV of surround view cannot cover the entire parking slot, only the nearby corner points can be detected, i.e., the width of the parking slot can be detected, and the length can only be inferred from priori rules. 

Three parking methods are adopted in the experiment: geometric method-based path planning with PID-based path tracking, geometric method-based path planning with SMC-based path tracking, and reinforcement learning-based end-to-end parking. The first two represent the current mainstream parking methods, and the last one represents the method used in this paper. Each parking method is at the same starting point and reversed at the same speed (4km/h).

**Figure 19:**
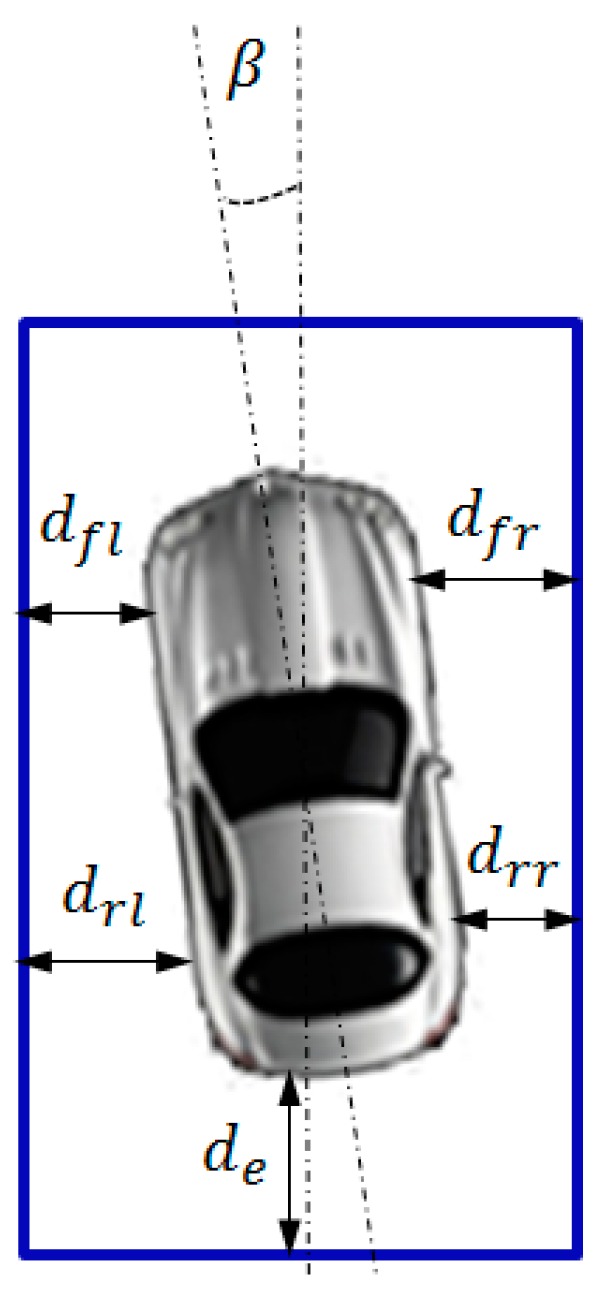
Measurement of parking attitude.

Subsequently, the parking performances of different parking methods are compared. According to BS ISO 16787-2016, the inclination angle of the vehicle with respect to the parking slot, the deviation between the four tire contact points of the vehicle and the parking slot, and the deviation between the rear of the vehicle and the parking slot are measured. The measurement parameters are presented in Figure 19.

### 3.3. Results

**Figure 20:**
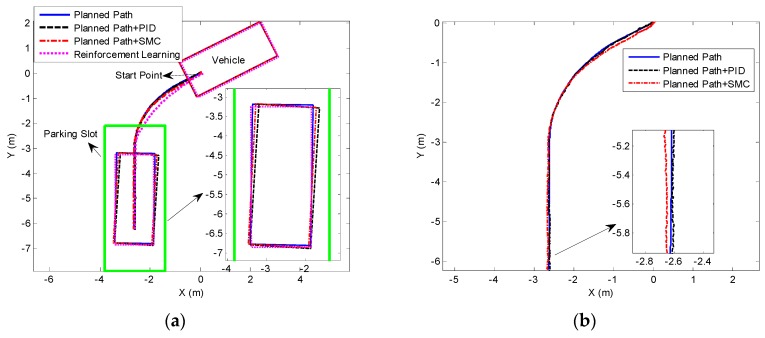

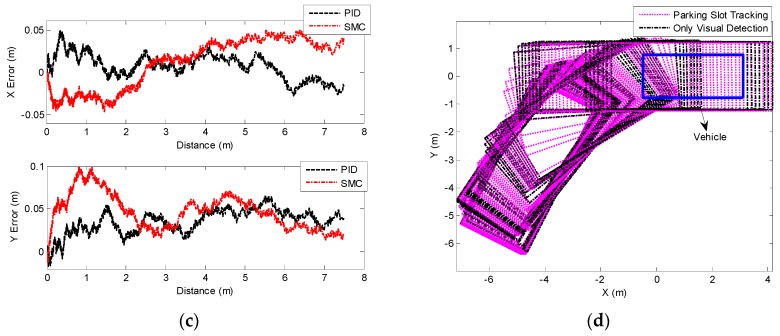
Experimental results of 60° perpendicular parking. (**a**) Parking performance of different methods; (**b**) and (**c**) present the control effect and error of different path tracking methods, respectively. (**d**) Parking slot detection and tracking in the parking process.

The experimental results of 60° perpendicular parking are presented in Figure 20. Figure 20b shows that only planned path can ensure the ideal parking attitude, whereas the two path tracking methods (PID and SMC) cannot completely track the planned parking path. The existing control error causes the final vehicle to deviate from the ideal parking attitude, as shown in Figure 20b,c. Figure 20b also shows that the parking performance of reinforcement learning is better than those of the other two methods. Besides, the changes of the parking slot in the vehicle CS are recorded in the case of only visual detection and parking slot tracking in the experiment of reinforcement learning, as shown in Figure 20d. It is suggested that visual detection has missed detection, and it cannot provide continuous parking slot for reinforcement learning. Thus, the test cannot be performed normally. However, the parking slot tracking has not missed detection.

The experimental data corresponding to Figure 20 is listed in Table 1. This table suggests that reinforcement learning can achieve an inclination angle of –0.747°, satisfying the requirements of the BS ISO 16787-2016 (≤±3°). Moreover, these deviations are relatively uniform, satisfying the requirements of the BS ISO 16787-2016 (>0.1 m). As mentioned above, only planned path can ensure that the ideal parking and inclination angle and deviation meet the requirements of the standard. However, when path tracking is practically performed, these deviations of path planning with PID and path planning with SMC are not uniform, and the inclination angles are –3.638° and –3.126°, respectively, which do not satisfy the requirements. These two path tracking methods have errors of more than 0.02 m in both X and Y directions. Lastly, it is suggested that the loss rate of visual detection is 37.35%, and that of the parking slot tracking reaches 0%.

**Figure 21:**
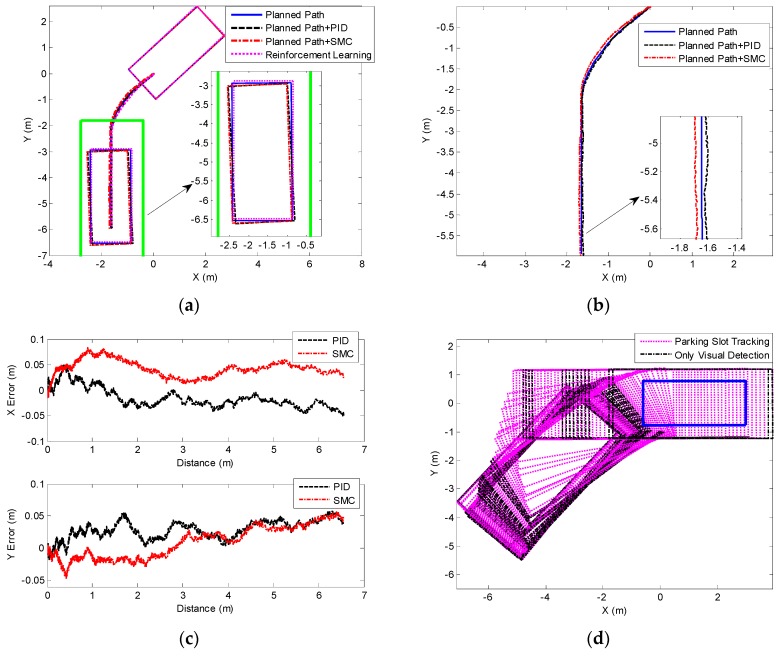
Experimental results of 45° perpendicular parking. (**a**) Parking performance of different methods; (**b**) and (**c**) represent the control effect and error of different path tracking methods, respectively. (**d**) Parking slot detection and tracking in the parking process.

**Figure 22:**
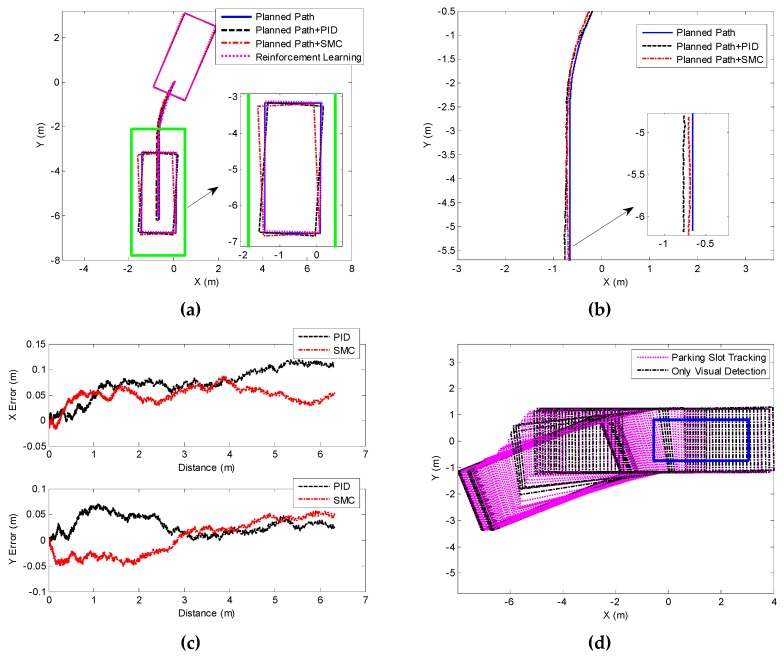
Experimental results of 30° perpendicular parking. (**a**) Parking performance of different methods; (**b**) and (**c**) present the control effect and error of different path tracking methods, respectively. (**d**) Parking slot detection and tracking in the parking process.

The experimental results of perpendicular parking at 45° and 30° initial angle are shown in Figure 21 and Figure 22. On the whole, the results are consistent with the 60° test. The parking performance of reinforcement learning is obviously superior over those of the other two methods, suggesting that our algorithm can adapt to parking scenario with different initial angles. Likewise, both PID and SMC have control errors, making it unlikely for the vehicle to track the parking path accurately, and eventually the vehicle has inclination angle and uniform deviation.

Table 2 and Table 3 suggest that reinforcement learning can achieve an inclination angle below 1°, satisfying the standard requirements. The other two methods have large inclination angle due to control error, especially the PID exceeding 3°. The path tracking errors of these two methods in X and Y directions are basically above 0.02 m. Besides, the loss rate of visual detection in these two scenarios reaches over 30%, and the parking slot tracking ensures that the position of the target parking slot can be continuously achieved.

## 4. Discussion

The above experimental results reveal that the existing mainstream parking methods of path planning with path tracking can basically park the vehicle into the parking slot, whereas the final inclination angle of the vehicle does not meet the strict requirements of the standard. This method is feasible for some wide parking slots. However, with the increasing number of vehicles, the design of parking slot will become narrower and narrower. Thus, the accuracy of parking should be enhanced. Besides, we can also see that it is not difficult to plan an ideal parking path according to the parking slot. However, due to the nonlinear dynamic characteristics of the vehicle, path tracking will inevitably produce control errors that cause the vehicle to deviate from the planned path, thereby resulting in inclination angle and uniform deviation of the parking attitude.

The reinforcement learning-based end-to-end planning method can not only achieve the end-to-end parking from parking slot to steering wheel angle, avoiding errors caused by path tracking, but also learn the best steering wheel angle through a lot of training. Thus, the reinforcement learning-based end-to-end planning can achieve better parking attitude. Besides, because we have fused the vision and vehicle chassis information, we can continuously get the position of parking slot to ensure the normal training and testing of reinforcement learning.

However, future research can still make some improvements: (1) The reward setting of this article is obtained by artificial setting and experimental adjustment. Though the final effect converges to an ideal level, it cannot be proved that it is the optimal reward setting. Accordingly, we will consider the method of inverse reinforcement learning [33,34] to optimize the reward. (2) In this paper, the reinforcement learning-based parking only has the function of reversing (e.g., “step two” in Figure 14), and it cannot automatically adjust the gear forward and backward. If the vehicle needs to judge the gear, we will consider selecting the Long Short-Term Memory (LSTM) network [35].

## 5. Conclusions

In this study, we innovatively adopt reinforcement learning to perpendicular parking so that the vehicle can continuously learn and accumulate experience from considerable parking attempts, learn the command of the optimal steering wheel angle at different parking slots relative to vehicle, as well as achieve real, “human-like” intelligent parking. Moreover, such end-to-end planning can avoid errors caused by path tracking. Besides, to ensure that the parking slot can be obtained continuously in the course of learning, a parking slot tracking algorithm is proposed based on fusion of vision and vehicle chassis information. Besides, since the learning network output is hard to converge and it is easy to fall into local optimum in the parking process, several reinforcement learning training methods in terms of parking conditions are designed (e.g., manual guided exploration for accumulating initial experience sequence, control cycle phased setting, and training condition phased setting). Lastly, the well-trained network in the simulation environment is migrated to the real vehicle training.

In the subsequent study, on one hand, inverse reinforcement learning will be used to set rewards to ensure optimal reward settings; on the other hand, the LSTM network will be used to achieve gear adjustment in the parking process.

## Figures and Tables

**Table 1:** Experimental data of 60° perpendicular parking.

	Planned Path	Planned Path+PID	Planned Path+SMC	Reinforcement Learning
Inclination angle *β* (°)	−1.051	−3.638	−3.126	−0.747
Deviation *d_fr_* (m)	0.423	0.304	0.379	0.457
Deviation *d*_*f*__l_ (m)	0.468	0.589	0.514	0.463
Deviation *d*_rr_ (m)	0.465	0.45	0.504	0.487
Deviation *d*_rl_ (m)	0.425	0.443	0.388	0.434
Deviation *d*_e_ (m)	1.087	1.016	1.047	1.053
X average error (m)	\	0.021	0.028	\
Y average error (m)	\	0.033	0.048	\
Loss rate of visual detection (%)	\	\	\	37.35
Loss rate of parking slot tracking (%)	\	\	\	0

**Table 2:** Experimental data of 45° perpendicular parking.

	Planned Path	Planned Path+PID	Planned Path+SMC	Reinforcement Learning
Inclination angle *β* (°)	0.313	3.088	2.011	−0.573
Deviation *d*_*fr*_ (m)	0.493	0.557	0.59	0.438
Deviation *d*_*f*l_ (m)	0.377	0.315	0.281	0.436
Deviation *d*_rr_ (m)	0.48	0.433	0.509	0.461
Deviation *d*_rl_ (m)	0.391	0.439	0.361	0.413
Deviation *d*_e_ (m)	0.872	0.788	0.795	0.918
X average error (m)	\	0.032	0.042	\
Y average error (m)	\	0.024	0.019	\
Loss rate of visual detection (%)	\	\	\	43.68
Loss rate of parking slot tracking (%)	\	\	\	0

**Table 3:** Experimental data of 30° perpendicular parking.

	Planned Path	Planned Path+PID	Planned Path+SMC	Reinforcement Learning
Inclination angle *β* (°)	−0.223	−3.782	2.416	−1.02
Deviation *d*_*fr*_ (m)	0.394	0.363	0.552	0.376
Deviation *d*_*f*l_ (m)	0.456	0.49	0.299	0.474
Deviation *d*_rr_ (m)	0.403	0.515	0.455	0.417
Deviation *d*_rl_ (m)	0.447	0.339	0.396	0.434
Deviation *d*_e_ (m)	1.031	0.952	0.948	1.056
X average error (m)	\	0.056	0.043	\
Y average error (m)	\	0.028	0.031	\
Loss rate of visual detection (%)	\	\	\	31.48
Loss rate of parking slot tracking (%)	\	\	\	0
